# Disability Mediates the Impact of Common Conditions on Perceived Health

**DOI:** 10.1371/journal.pone.0065858

**Published:** 2013-06-06

**Authors:** Jordi Alonso, Gemma Vilagut, Núria D. Adroher, Somnath Chatterji, Yanling He, Laura Helena Andrade, Evelyn Bromet, Ronny Bruffaerts, John Fayyad, Silvia Florescu, Giovanni de Girolamo, Oye Gureje, Josep Maria Haro, Hristo Hinkov, Chiyi Hu, Noboru Iwata, Sing Lee, Daphna Levinson, Jean Pierre Lépine, Herbert Matschinger, Maria Elena Medina-Mora, Siobhan O'Neill, J. Hormel, Jose A. Posada-Villa, Nezar Ismet Taib, Miguel Xavier, Ronald C. Kessler

**Affiliations:** 1 IMIM-Institut Hospital del Mar d'Investigacions Mèdiques, Barcelona, Spain; 2 Pompeu Fabra University, Barcelona, Spain; 3 CIBER en Epidemiología y Salud Pública, Barcelona, Spain; 4 World Health Organization, Geneva, Switzerland; 5 Shangai Mental Health Center, Shangai, People's Republic of China; 6 Institute of Psychiatry University of São Paulo Medical School, São Paulo, Brazil; 7 State University of New York at Stony Brook, Stony Brook, New York, United States of America; 8 Universitair Psychiatrisch Centrum - Katholieke Universiteit Leuven, Leuven, Belgium; 9 Institute for Development Research, Advocacy, and Applied Care, Beirut, Lebanon; 10 St. George Hospital University Medical Center, Beirut, Lebanon; 11 National School of Public Health Management and Professional Development, Bucharest, Romania; 12 IRCCS Centro S. Giovanni di Dio Fatebenefratelli, Brescia, Italy; 13 University College Hospital, Ibadan, Nigeria; 14 Parc Sanitari Sant Joan de Déu, Sant Boi de Llobregat, Barcelona, Spain; 15 CIBERSAM, Sant Boi de Llobregat, Barcelona, Spain; 16 National Center for Public Health Protection, Sofia, Bulgaria; 17 Shenzhen Institute of Mental Health and Shenzhen Kangning Hospital, Guangdong Province, People's Republic of China; 18 Hiroshima International University, Higashi-Hiroshima, Japan; 19 The Chinese University of Hong Kong, Shatin, HKSAR; 20 Ministry of Health, Jerusalem, Israel; 21 Hôpital Saint-Louis Lariboisière Fernand Widal, Paris, France; 22 Universität Leipzig, Leipzig, Germany; 23 Instituto Nacional de Psiquiatria Ramon de la Fuente, Mexico City, Mexico; 24 University of Ulster, Londonderry, United Kingdom; 25 University Medical Center Groningen, Groningen, The Netherlands; 26 Pontificia Universidad Javeriana, Bogota D.C., Colombia; 27 Mental Health Center-Duhok, Kurdistan Region, Iraq; 28 Universidade Nova de Lisboa, Lisbon, Portugal; 29 Harvard Medical School, Boston, Massachusetts, United States of America; The University of Hong Kong, Hong Kong

## Abstract

**Background:**

We examined the extent to which disability mediates the observed associations of common mental and physical conditions with perceived health.

**Methods and Findings:**

WHO World Mental Health (WMH) Surveys carried out in 22 countries worldwide (n = 51,344 respondents, 72.0% response rate). We assessed nine common mental conditions with the WHO Composite International Diagnostic Interview (CIDI), and ten chronic physical with a checklist. A visual analog scale (VAS) score (0, worst to 100, best) measured perceived health in the previous 30 days. Disability was assessed using a modified WHO Disability Assessment Schedule (WHODAS), including: cognition, mobility, self-care, getting along, role functioning (life activities), family burden, stigma, and discrimination. Path analysis was used to estimate total effects of conditions on perceived health VAS and their separate direct and indirect (through the WHODAS dimensions) effects.

Twelve-month prevalence was 14.4% for any mental and 51.4% for any physical condition. 31.7% of respondents reported difficulties in role functioning, 11.4% in mobility, 8.3% in stigma, 8.1% in family burden and 6.9% in cognition. Other difficulties were much less common. Mean VAS score was 81.0 (SD = 0.1). Decrements in VAS scores were highest for neurological conditions (9.8), depression (8.2) and bipolar disorder (8.1). Across conditions, 36.8% (IQR: 31.2–51.5%) of the total decrement in perceived health associated with the condition were mediated by WHODAS disabilities (significant for 17 of 19 conditions). Role functioning was the dominant mediator for both mental and physical conditions. Stigma and family burden were also important mediators for mental conditions, and mobility for physical conditions.

**Conclusions:**

More than a third of the decrement in perceived health associated with common conditions is mediated by disability. Although the decrement is similar for physical and mental conditions, the pattern of mediation is different. Research is needed on the benefits for perceived health of targeted interventions aimed at particular disability dimensions.

## Introduction

Perceived or self-rated health is widely recognized as an important indicator of health [1], [2] and is often used to monitor health trends in the general population [3] as well as to assess patient-centered outcomes in clinical studies [4]. Although the need to go beyond an exclusive focus on perceptions has been pointed out [5], [6], perceived health is nonetheless an important indicator variable that has been shown to predict mortality independently of the presence and severity of disease and risk factors [7], as well as to predict health services utilization and health care costs [8], and future disability [9], [10].

Chronic conditions are among the most important predictors of perceived health [11]–[15]. Some conditions, such as those causing pain, are known to be associated with great decrements in perceived health [16]. We previously reported important decrements in perceived health associated with neurological conditions, depression and arthritis once the presence of other conditions had been taken into account [17]. A higher impact of mental conditions (as compared to other medical conditions) on perceived health has also been reported [18].

Prevalent conceptual frameworks and models of health propose that disability mediates the impact of chronic conditions on perceived health [19]–[21]. A mediation model proposes a causal mechanism of relation between an independent variable (i.e., chronic conditions) which has an effect on a third explanatory variable, the mediator (i.e., disability), which in turn influences an outcome (i.e., perceived health) [22]–[23]. Consistent with this model, mounting evidence shows that disability is significantly associated with perceived health both cross-sectionally [14], [24] and longitudinally [15], [24], [25]. There is also evidence that chronic conditions are significantly associated with disability [26]–[28]. A few studies have assessed the mediating role of disability in the association of chronic conditions and mental health [29], [30]. However, we are not aware of any systematic attempt to identify the extent to which different dimensions of disability mediate the overall associations of chronic conditions with perceived health in epidemiological samples. Such an analysis could have value in enhancing our understanding of the pathways that link chronic conditions to perceived health. In turn, this could help customizing condition-specific interventions aimed at ameliorating the disabilities that lead to significant decrements in perceived health.

In this paper we explore the extent to which a multidimensional assessment of disability mediates the associations of 19 chronic conditions (9 mental, 10 physical) on perceived health in surveys of the WHO World Mental Health (WMH) surveys initiative [31], a consortium of cross-sectional general population epidemiological surveys carried out in 22 developing and developed countries throughout the world. We focus not only on the extent to which disability mediates the total effects of each condition, but also on the relative importance of individual disability dimensions and how it varies across type of conditions. We had hypothesized that a significant proportion of the decrease in perceived health status associated to mental and physical conditions would be mediated by specific disability dimensions. We also anticipated that the pattern of disability mediation (i.e., the portion of the effect of the chronic condition on perceived health VAS score that was explained by its association with the disability dimensions and the association of the latter with perceived health) could be different for mental and for physical conditions.

## Methods

### Sample

A total of 23 surveys were carried out in 22 countries, 6 classified by the World Bank (2009), at the time of data collection, as low and lower-middle income (Colombia, India (Pondicherry region), Iraq, Nigeria, Peoples' Republic of China (cities of Beijing/Shanghai and Shenzhen), and Ukraine), 5 upper-middle income countries (Brazil –Sao Paulo metropolitan area-, Bulgaria, Lebanon, Mexico and Romania) and 11 high income (Belgium, France, Germany, Israel, Italy, Japan, Netherlands, Northern Ireland, Portugal, Spain, and United States of America). Informed consent was obtained before beginning interviews, using procedures approved by the institutional review board of the organization coordinating the survey in each country (please, see additional online table). The weighted average response rate across countries was 72.0%, with country-specific response rates ranging from 45.9% (France) to 87.7% (Colombia). All surveys were based on probability samples of the country's adult household population that were either nationally representative (in the majority of countries) or representative of particular regions of the country (in China, Colombia, India, Japan, and Mexico). The age ranges of the sample varied across participating countries. Most countries had a minimum age of 18 years, while the minimum in Japan and Israel were 20 and 21, respectively. The upper age was unrestricted in most surveys but was 70 in China and 65 in Colombia and Mexico. Additional details about sampling and respondents are available elsewhere.

All interviews were conducted face-to-face by trained lay interviewers either using a computer assisted personal interview (CAPI) or a paper and pencil interview (PAPI). In most of the countries, except Iraq, Romania and Israel, each interview had two parts. All respondents completed Part 1, which contained core mental conditions, while all Part 1 respondents who met criteria for any core mental condition plus a random probability sub-sample of other Part 1 respondents were administered Part 2 (the latter assessing, in detail, correlates, service use, and conditions of secondary interest to the study). Data were weighted to adjust for differential probabilities of selection and to match population distributions on socio-demographic and geographic data. An additional weight was used for the over sampling of respondents for the Part 2 sample.

Updated WHO guidelines for translation and back-translations focusing on conceptual equivalence were used for all study materials. Pretesting and independent experts' evaluations indicated equivalent measures. Certified lay interviewers were used for data collection, since they tend to achieve highly reliable measures [32]. Standardized procedures for interviewer training were followed in all settings including a certification process and a close supervision of data quality. These procedures are described in more detail elsewhere [33]. Informed consent was obtained from all respondents. Procedures for obtaining informed consent and protecting human subjects were approved and monitored for compliance by the Institutional Review Boards of the organizations coordinating the surveys in each country.

### Chronic physical conditions

Physical conditions were assessed with a standard chronic conditions checklist that asked respondents if they had ever suffered from the given physical health condition, if they had the condition in the past 12 months and if they had received any treatment. Such checklists have been shown to provide more accurate and complete self-reports than as compared to open-ended questions. Methodological studies suggest a moderate to good concordance between such reports and medical records [34], [35].

The ten conditions considered here are: arthritis, cancer, cardiovascular (heart attack, heart disease, hypertension, and stroke), chronic pain (chronic back or neck pain and other chronic pain), diabetes, frequent or severe headache or migraine, insomnia, neurological (multiple sclerosis, Parkinson's, and epilepsy or seizures), digestive condition (stomach or intestine ulcer or irritable bowel condition), and respiratory (seasonal allergies like hay fever, asthma, or COPD or emphysema). For the symptom based conditions like arthritis, chronic pain and headache, heart attack or stroke respondents were asked to report whether they had experienced these conditions. For the remaining silent conditions the question was prefaced by the phrase “*have you ever been told by a doctor or health professional that you had any of these conditions?”* The time frames varied across countries and chronic conditions: the western European countries assessed both lifetime and 12-month presence of each condition, while for the rest of the countries that used the CAPI version of the questionnaire some of the chronic conditions were only evaluated lifetime, but for problems that could have remitted, participants were asked if they still had the conditions in the past 12 months. Finally, the PAPI countries used a 12 month time frame for most symptom-based conditions and lifetime (LT) frame for the silent conditions. The 12 month time frame has been used whenever possible but, for some of the conditions inconsistent time frames were used across countries. Generally good agreement between self-report of medical diagnoses and physician or medical record confirmation of those diagnoses [32], [33].

### Mental conditions

Mental conditions were assessed with Version 3.0 of the World Health Organization (WHO) Composite International Diagnostic Interview (CIDI 3.0), a fully structured lay-administered interview designed to generate diagnoses of mental conditions based on the Diagnostic and Statistical Manual of the American Psychiatric Association, IV^th^ edition (DSM-IV). The mental conditions considered here are: depressive conditions (major depressive condition, minor depressive condition), bipolar disorder (mania, hypomania, bipolar I, bipolar II), panic disorder (Panic disorder, agoraphobia without panic), specific phobia, social phobia, generalized anxiety condition, post-traumatic stress disorder, alcohol abuse with and without dependence, drug abuse with and without dependence.

Only conditions present in the past 12 months are considered in this report. Generally good concordance has been found between CIDI diagnoses of anxiety and depressive conditions and independent clinical assessment [36], [37].

### Perceived health

Overall perceived health was assessed using a visual analog scale (VAS) approach [17]. Respondents were asked to use a 0 to 100 scale where 0 represents the worst possible health a person can have and 100 represents perfect health to *“describe your own overall physical and mental health during the past 30 days”* taking into consideration all the physical and mental conditions reviewed in the survey.

### Disability

Disability was assessed with a modified version of the WHO Disability Assessment Schedule 2.0 (WHODAS) [38], [39]. Questions were asked about difficulties in: a) understanding and communication (cognition), b) moving and getting around (mobility), c) attending personal hygiene, dressing and eating, and living alone (self-care) and d) interacting with other people (getting along). In addition, a series of questions about activity limitations days replaced the WHODAS life activities domain questions. In these questions, respondents were asked the number of days out of the past 30 that they were totally unable to carry out their normal activities or work; that they had to cut down in the activities; that they had to reduce their quality; or that they needed to exert an extreme effort to carry out their activities, due to physical or mental health problems (role functioning). Respondents were also asked about the extent of embarrassment (stigma) and discrimination or unfair treatment (discrimination) they experienced due to their health condition and, finally, they were asked about the interference of their health condition on the day to day activities of their family members (family burden). Scores on each dimension were computed, ranging from 0 to 100, where 0 indicated no disability and 100 indicated complete disability.

### Statistical analysis

We used SUDAAN V10.0 (RTI International, USA) to generate estimates of condition prevalence and descriptive statistics for the distributions of the continuous variables. We then used MPlus 6.0 (Muthén and Muthén, Los Angeles, CA) to conduct all multivariate analyses in parallel in the total sample and within three subsamples consisting of respondents low and lower-middle, upper-middle, and high income countries.

**Figure 1:**
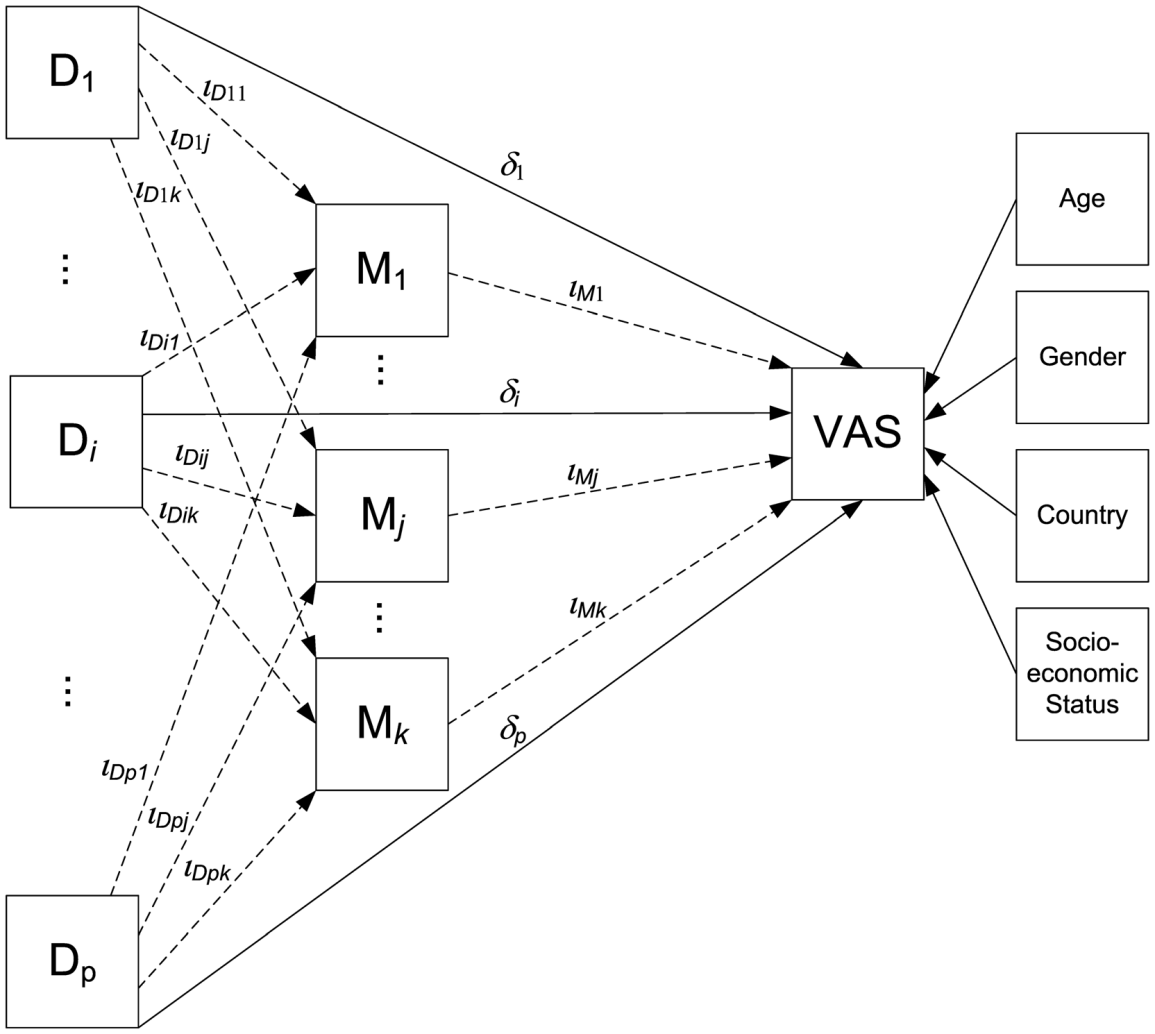
General mediation model used in analyses. The figure displays the general mediation model that has been used to estimate effects according to path-diagrammatic conventions. Squares represent variables. *D_i_* is one of the *p = 19* disorders under consideration, *M_j_* is one of the *k = 8* mediating variables (disability dimensions), and VAS is the final outcome. Arrows represent regression slope parameters from independent variables to outcomes. The *δ* parameters stand for the direct effect regression from disorders to the final outcome. The *ι* parameters indicate the two regression components of the disorder indirect effects as mediated by *M*: a) *p* x *k* regression parameters from *D* to *M* (*ι_Dij_*) and b) k regression parameters from *M* to VAS (*ι_M_*_j_). For each disorder the model can be decomposed in two paths: 1) VAS regressed on disorders, and 2) a causal mediation chain of VAS regressed on mediators which in turn are regressed on disorders. The partial indirect effect of a certain disorder *D_i_* through a mediator *M_j_* is *ι_Dij_* x *ι_Mj_*, whereas its total indirect effect is the sum of the *k* products across mediators (
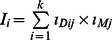
). Total effects for a disorder are the sum of direct and total indirect effect (*δ_i_*+*Ι_i_*). Directionality cannot be assumed as a causal association in our study due to its cross-sectional, observational nature. Also notice that in the general model, the effect of each disorder on each mediator is adjusted by the direct effect of the remaining disorders (thus controlling for comorbidity), while the impact of a disorder on VAS is controlled by the total effects of the other disorders. Disability is thus fully taken into account, even though it is decomposed in subscales. The effects on VAS are also controlled for age, gender, employment status and country.

Path analysis was used to estimate, through simultaneous regression mediation submodels, the total, the direct, and the indirect (i.e., mediated by disability) effects of each condition in predicting VAS scores. The direct effect of each condition on perceived health VAS score is that part of its total effect which is not mediated via intervening variables. The indirect effects of each condition on VAS, via WHODAS domains, were generated as the product of regression coefficients (the regression coefficient of VAS score regressed on the WHODAS domain multiplied by the coefficient of the domain regressed on the condition). The submodels of the path analysis were embedded in a single general structural model as detailed in **Figure 1** (see figure legend). Note that in the general model the effects of each disorder on the mediator variables are controlled by the direct effect of the remaining disorders (thus adjusting for comorbidity) and the VAS is adjusted by the total effects of all disorders as well as sociodemographic variables (age, gender, employment status and country). The final model took into account 19 disorders and 8 mediating dimensions. For purposes of comparing the relative magnitude of the direct and indirect effects across conditions, the total effect within condition (i.e. the sum of the direct effect plus all indirect effects) was rescaled to sum up 100%. To estimate model parameters, we used the maximum likelihood estimation method. To account for the complex sample design, standard errors and statistical tests were calculated using a sandwich estimator implemented in M-PLUS, which is equivalent to the Taylor series linearization method.

## Results

A total of 51,344 respondents (Part 2 respondents) were assessed of which 16,051 were from low/lower-middle income, 10,496 from upper-middle income, and 24,797 from high income countries (Table 1). Individuals had an average of 42 years of age, varying from an average of 37 in low/lower-middle income to an average of 46 in the high income countries. Almost 52% were female and just above a third (35.7%) were not married. The proportion of individuals with completed high school varied from 47.2% in the low/lower-middle income to 71.8% in the high income countries. Overall, 41.6% of the sample was not working (41.9% in low/lower-middle income, 46.5% in upper-middle income and 39.3% in high income countries).

**Table 1:** Sample Characteristics per country income level. The WMH Surveys.

Country	N	Females	Not Married	= >High School	Not Working	Age	Any Mental	Any Physical	Any Mental or Physical
		% (SE)	% (SE)	% (SE)	% (SE)	Mean (SE)	% (SE)	% (SE)	% (SE)
**Low and Lower-middle income**									
Colombia	2381	54.5 (1.5)	43.4 (1.7)	46.4 (2.3)	46.4 (2)	36.6 (0.3)	18.7 (1)	42.7 (1.3)	50.4 (1.5)
India - Pondicherry	1373	50 (1.6)	30.2 (2.1)	47 (1.9)	52.1 (1.5)	38.1 (0.6)	19 (1)	40.7 (2)	47.2 (2.1)
Iraq	4332	49.7 (1)	34.4 (1.2)	35.3 (1.1)	59.2 (1.2)	36.9 (0.4)	10.9 (0.7)	44.5 (1.1)	47.8 (1)
Nigeria	2143	51 (1.5)	39.7 (1.6)	35.6 (1.3)	31.1 (1.4)	35.8 (0.4)	6.3 (0.6)	46.2 (1.6)	49.2 (1.6)
PRC –Beijing/Shanghai	1628	47.7 (1.8)	33.4 (1.7)	55 (1.6)	41.2 (2.2)	41.2 (0.6)	6 (0.8)	53 (1.8)	54.8 (1.8)
PRC – Shenzhen	2475	50.3 (1.6)	46.2 (1.3)	49.4 (1.5)	8.5 (0.6)	29.1 (0.3)	7.8 (0.7)	30.1 (1.2)	33.8 (1.2)
Ukraine	1719	55.1 (1.3)	34.9 (1.4)	81.9 (1.5)	45.6 (2.1)	46.1 (0.8)	20.4 (1.3)	71.3 (1.8)	75 (1.8)
**Upper-Middle income**									
Brazil	2942	52.8 (1.5)	40.2 (1.6)	47.2 (1.3)	35.4 (1.1)	39.1 (0.5)	27.3 (0.8)	68.5 (2)	73.5 (1.7)
Bulgaria	2233	52.2 (1.3)	25.7 (1.6)	64.2 (1.3)	50.4 (1.9)	47.8 (0.6)	10.7 (0.6)	44.3 (1.3)	48.1 (1.4)
Lebanon	602	48.1 (2.6)	39 (3.2)	40.5 (2.8)	48.9 (2.4)	40.3 (0.9)	10 (1.5)	38.8 (2.1)	43.8 (2.5)
Mexico	2362	52.3 (1.9)	32.7 (1.5)	31.4 (1.7)	41.6 (1.6)	35.2 (0.3)	12.7 (0.8)	35 (1.8)	40.7 (1.8)
Romania	2357	52.4 (1.3)	30.4 (1.2)	49.3 (1.7)	60.7 (1.3)	45.5 (0.5)	7 (0.6)	48.1 (1.3)	50.6 (1.3)
**High income**									
Belgium	1043	51.7 (2.4)	30.2 (1.7)	69.7 (3.7)	42.2 (1.4)	46.9 (0.7)	13.8 (1.7)	48.8 (2.2)	54.1 (2.4)
France	1436	52.2 (1.8)	29 (1.8)	. (.)	37.9 (1.8)	46.3 (0.7)	18.5 (1.3)	52.7 (2.2)	59.5 (2.3)
Germany	1323	51.7 (1.4)	36.7 (1.7)	96.4 (0.9)	43.5 (2.1)	48.2 (0.8)	10.9 (1.3)	49.7 (2.4)	54.2 (2.4)
Israel	4859	51.9 (0.4)	32.2 (0.7)	78.3 (0.7)	39.8 (0.8)	44.4 (0.2)	10.7 (0.5)	55.5 (0.8)	58 (0.8)
Italy	1779	52 (1.5)	33.3 (1.6)	39.4 (1.8)	46.1 (1.7)	47.7 (0.6)	8.9 (0.7)	50 (1.8)	52.9 (1.7)
Japan	1682	53 (1.9)	31.2 (1.4)	71.6 (1.4)	36.5 (1.8)	51.2 (0.7)	7.6 (0.6)	52.8 (1.8)	55.6 (1.9)
Netherlands	1094	50.9 (2.2)	27.9 (2.6)	69.7 (1.8)	37.7 (2.6)	45 (0.8)	13.5 (1)	48.5 (2.3)	52.8 (2.2)
N.Ireland	1708	51 (1.4)	40.4 (1.8)	88.7 (1)	37.4 (1.9)	45.3 (0.6)	19.1 (1.5)	54.4 (1.9)	60.6 (1.8)
Portugal	2060	51.9 (1.5)	30.4 (1.4)	54.8 (1.7)	40.3 (1.5)	46.5 (0.7)	21.8 (0.9)	55.1 (1.6)	63.1 (1.6)
Spain	2121	51.4 (1.7)	34.7 (1.5)	41.7 (1.5)	49.6 (1.8)	45.6 (0.7)	9.9 (0.9)	42.8 (1.5)	47.1 (1.5)
United States	5692	53.1 (1)	44.1 (1.2)	83.2 (0.9)	33.2 (1.1)	45 (0.5)	24.5 (0.8)	70.1 (1)	75 (1)
All countries	51344	51.8 (0.3)	35.7 (0.3)	58.6 (0.4)	41.6 (0.3)	42.3 (0.1)	14.4 (0.2)	51.4 (0.4)	55.8 (0.4)
comparison among countries		1.2 (0.2)	13.8 (<0.0001)	165.6 (<0.0001)	63 (<0.0001)	187.9 (<0.0001)	59.8 (<0.0001)	38.6 (<0.0001)	40.4 (<0.0001)
High income	24797	52.1 (0.4)	35.4 (0.5)	71.8 (0.5)	39.3 (0.5)	46 (0.2)	15.7 (0.3)	56 (0.5)	60.5 (0.5)
Upper-Middle income	10496	52.1 (0.7)	33.3 (0.7)	47.2 (0.8)	46.5 (0.7)	41.5 (0.2)	14.8 (0.4)	49.3 (0.8)	53.6 (0.7)
Low and Lower-middle income	16051	51.1 (0.6)	37.9 (0.6)	47.2 (0.6)	41.9 (0.6)	37 (0.2)	12.1 (0.3)	45.6 (0.6)	49.8 (0.6)
comparison low, middle, high		1.2 (0.3)	12.6 (<0.0001)	582.2 (<0.0001)	36.9 (<0.0001)	633 (<0.0001)	31.4 (<0.0001)	85.5 (<0.0001)	89.6 (<0.0001)

Physical chronic conditions were more prevalent than mental conditions, with 12 month prevalence ranging from a lowest of 30.1% (Shenzhen, China) to a highest of 71.3% in Ukraine and 70.1% in the United States. The prevalence of mental conditions ranged from a lowest of 6% in Beijing/Shanghai (China) to a highest of 27.3% in Sao Paulo metropolitan area (Brazil) and 24.5% in the United States. There was a trend towards higher prevalence of conditions among in higher income countries.

Chronic pain was the most common condition in low/lower-middle income (21.9%), in upper-middle income (20.5%), and in high income (21.6%) countries. In the latter, cardiovascular conditions (19.3%) were also very common. Other common physical conditions in all countries were respiratory, cardiovascular, arthritis and headache/migraine. The prevalence of any physical condition ranged from 56% in high income to 45.6% in low/lower-middle income countries. Any mental condition ranged from 15.7% (high income) to 12.1%, (low/lower middle income) (data not shown but available upon request).

### Distribution of WHODAS scores

Table 2 shows the proportion of respondents with difficulties on each of the WHODAS dimensions for the overall sample and for each income country category. Over a third of respondents (35.7%) had some difficulty in the WHODAS (score>0), the frequency being considerably higher among respondents from high income countries (46.5%) than for those in other countries (22.2% and 28%). Role functioning dimension was the most frequently affected dimension (31.7%) in all income country categories (from 42% to 18.3%). Mobility and stigma showed the second most frequent difficulties (11.4% and 8.3%, respectively in the overall sample), while self-care was the least frequently affected (3.4%).

**Table 2:** Distribution of the WHODAS dimension scores by income level. The WMH Surveys.

			Across non-zero			
	% with non-zero score (SE)	Mean across all (SE)	Mean (SE)	p25	median	p75
**Overall sample**						
Cognition	6.9 (0.14)	0.8 (0.03)	12 (0.32)	1.7	5	15.6
Mobility	11.4 (0.19)	3.2 (0.07)	28.2 (0.48)	5	16.7	50
Self-care	3.4 (0.1)	1 (0.05)	28.3 (1.12)	5	16.1	50
Getting along	3.9 (0.11)	0.6 (0.03)	15.8 (0.54)	2.3	7.5	22.2
Role functioning	31.7 (0.3)	9 (0.14)	28.5 (0.36)	3.2	10.8	46.7
Family burden	8.1 (0.15)	3.7 (0.08)	45.2 (0.46)	25	50	50
Stigma	8.3 (0.16)	4 (0.08)	48.7 (0.44)	25	50	75
Discrimination	3.5 (0.1)	1.7 (0.05)	47.1 (0.69)	25	50	50
Global Whodas	35.7 (0.3)	2.9 (0.05)	8.2 (0.12)	0.6	2.5	10
**High income countries**						
Cognition	7.9 (0.2)	1 (0.04)	12.5 (0.4)	1.7	5	16.7
Mobility	14.6 (0.3)	4.4 (0.13)	30.3 (0.64)	5	19.4	53.3
Self-care	4.1 (0.15)	1.3 (0.08)	30.3 (1.5)	5	16.7	50
Getting along	4.8 (0.17)	0.8 (0.04)	15.6 (0.59)	2.3	7.5	21.7
Role functioning	42 (0.43)	10.7 (0.21)	25.5 (0.45)	3.2	6.7	37.5
Family burden	8.7 (0.22)	3.9 (0.11)	45.2 (0.65)	25	50	50
Stigma	7.6 (0.19)	3.6 (0.1)	46.8 (0.68)	25	50	50
Discrimination	2.8 (0.11)	1.3 (0.06)	48.2 (1.05)	25	50	75
Global Whodas	46.5 (0.43)	3.6 (0.08)	7.8 (0.15)	0.6	1.7	10
**Upper-middle income countries**						
Cognition	5.8 (0.3)	0.8 (0.06)	13.3 (0.79)	1.9	5	18.3
Mobility	7.8 (0.3)	2.2 (0.11)	28.8 (1.01)	5.6	16.7	50
Self-care	2.1 (0.17)	0.7 (0.06)	31.2 (2.39)	6.7	16.7	50
Getting along	2.2 (0.18)	0.5 (0.06)	22.8 (1.84)	4	15	36.7
Role functioning	18.3 (0.56)	7.1 (0.27)	38.8 (1.21)	8.3	25	60
Family burden	7.7 (0.27)	3.5 (0.14)	45.3 (0.9)	25	50	50
Stigma	9.1 (0.32)	4.8 (0.18)	52.3 (0.76)	25	50	75
Discrimination	4.3 (0.2)	2 (0.11)	47.3 (1.17)	25	50	75
Global Whodas	22.2 (0.58)	2.3 (0.09)	10.1 (0.35)	1.3	5	13.3
**Low and lower-middle income countries**						
Cognition	6.1 (0.27)	0.6 (0.05)	10.4 (0.68)	1.5	4.4	12.5
Mobility	8.7 (0.29)	2 (0.09)	22.4 (0.86)	3.9	11.1	33.3
Self-care	3.2 (0.2)	0.7 (0.09)	22.9 (2.16)	3.3	10	32.5
Getting along	3.5 (0.2)	0.5 (0.05)	13.1 (1.16)	2	5.3	16.7
Role functioning	24.6 (0.46)	7.8 (0.23)	31.6 (0.69)	6.7	16.7	49.2
Family burden	7.4 (0.28)	3.3 (0.15)	45.2 (0.92)	25	50	50
Stigma	8.9 (0.35)	4.3 (0.18)	48.7 (0.82)	25	50	75
Discrimination	4.3 (0.23)	1.9 (0.11)	45.8 (1.26)	25	50	50
Global Whodas	28 (0.48)	2.3 (0.08)	8.2 (0.23)	1.3	3.5	10.3

Table 2 also shows the mean scores in each WHODAS dimension and the global score. For the latter, mean scores were higher for high income countries (3.6) than for upper-middle and low and lower-middle income countries (2.3). But mean global WHODAS scores across those with any difficulty tended to be higher for upper-middle income (10.1) and low/lower-middle income (8.2) than for high income countries (7.8).

### Distribution of perceived health VAS score

As shown in more detail in a previous WMH report [17], the mean VAS score was 81.0 in the overall sample. Respondents with mental conditions showed lower mean perceived health (72.2) than those with physical conditions (75). As shown in Table 3, these trends are consistent across all country income groups.

**Table 3:** Perceived health visual analogue scale (VAS) scores by country income level.

	All countries				High				Middle				Low			
	Mean (SE)	Q25	Median	Q75	Mean (SE)	Q25	Median	Q75	Mean (SE)	Q25	Median	Q75	Mean (SE)	Q25	Median	Q75
Overall sample	81 (0.1)	70	90	95	80.7 (0.2)	74.4	89.82	90	81 (0.3)	70	89.85	99.5	81.6 (0.2)	70	90	99.8
Any Mental condition	72.2 (0.3)	59.9	79.94	89.9	72.6 (0.4)	60	79.9	89.9	71.6 (0.7)	59.1	79.94	89.7	71.9 (0.7)	59.8	79.06	89.2
Any Physical condition	75 (0.2)	60	79.96	90	76 (0.2)	69.1	79.95	89.7	73.2 (0.5)	60	79.95	89.9	74.3 (0.4)	59.9	79.87	90
Any Mental or Physical condition	75.5 (0.2)	64.9	79.97	90	76.4 (0.2)	69.2	79.96	89.9	73.8 (0.5)	60	79.96	89.9	74.9 (0.4)	60	79.88	90

The WMH Surveys.

### Direct and indirect (disability mediated) effects of conditions on perceived health

**Figure 2:**
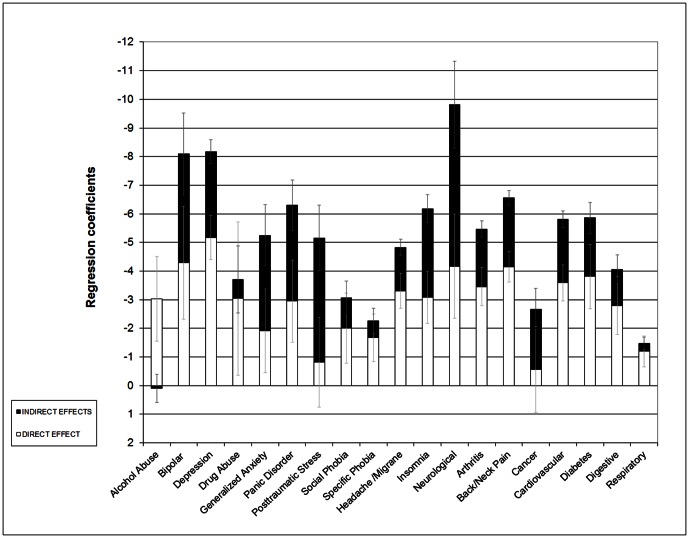
Direct and indirect effects (via WHODAS dimensions) of common chronic conditions on perceived health VAS, overall sample. WMH Surveys.

Table 4 presents the association of mental conditions and chronic physical conditions with VAS score for the overall sample. Total effects are highest for neurological conditions, with an average decrement of 9.8 points on the VAS, depression (8.2) and bipolar disorder (8.1). There is considerable variation across conditions in the extent to which total effects are mediated by WHODAS scores. The fourth column shows the proportion of overall indirect effects to total effects. Indirect effects tend to represent a lower proportion (among significant percentages in column 4, ranging from 19.4% to 84.0%, with a median of 36.8%, IQR = 31.2 to 51.5) of the total effect of the conditions on the VAS. Of notice some effects are non significant. These proportions can be visualized in Figure 2, where the total effect of each condition on the VAS is broken down into direct (shown in white) and indirect (in black) effects. In general, mental conditions tend to show higher proportions of indirect effects mediated by disability dimensions, with the highest values for PTSD (84.0%), GAD (63.7%), panic (53.1%), and bipolar disorder (47.0%). The chronic physical conditions with the highest proportions of total effects mediated by disability include cancer (78.9%), neurological conditions (57.6%), and insomnia (50.0%). Alcohol abuse and drug abuse are the only conditions considered here for which indirect effects through WHODAS scores are not statistically significant. Once adjusted by the remaining WHODAS dimensions and disorders, the dimensions most often associated with significant mediating effects across the 19 conditions are role functions (89.5%), family burden (84.2%), stigma (79.0%), mobility (73.7%), cognition (68.4%), and self-care (42.1%).

**Table 4:** Effects (direct and indirect via WHODAS dimension scores) of conditions on perceived health VAS, overall sample.

					Indirect effects via each WHODAS dimension^1^					
	Total effects of conditions on VAS	Direct effects of conditions	Indirect effects via WHODAS Scales	Percentage of indirect effects over total effects	Cognition	Mobility	Self-care	Role functioning	Family burden	Stigma
	Coeff (SE)	Coeff (SE)	Coeff (SE)	% (SE)	Coeff (SE)	Coeff (SE)	Coeff (SE)	Coeff (SE)	Coeff (SE)	Coeff (SE)
Alcohol Abuse	−2.94 (0.8)*	−3.03 (0.76)*	0.09 (0.25)	−3.16 (8.83)	0 (0.02)	0.32 (0.06)*	0.03 (0.02)	−0.11 (0.11)	−0.04 (0.06)	−0.1 (0.08)
Bipolar	−8.09 (1.28)*	−4.29 (1.01)*	−3.8 (0.73)*	47.02 (7.38)*	−0.48 (0.13)*	−0.26 (0.16)	−0.1 (0.08)	−1.22 (0.24)*	−0.86 (0.19)*	−0.77 (0.2)*
Depression	−8.17 (0.42)*	−5.17 (0.4)*	−3 (0.21)*	36.75 (2.6)*	−0.27 (0.06)*	−0.31 (0.07)*	−0.05 (0.02)*	−1.08 (0.1)*	−0.54 (0.08)*	−0.68 (0.09)*
Drug Abuse	−3.71 (1.49)*	−3.04 (1.37)*	−0.67 (0.6)	17.98 (14.74)	−0.09 (0.06)	0.13 (0.1)	0.03 (0.02)	−0.32 (0.22)	−0.28 (0.2)	−0.09 (0.17)
Generalized Anxiety	−5.25 (0.93)*	−1.91 (0.75)*	−3.34 (0.55)*	63.66 (9.77)*	−0.28 (0.08)*	−0.49 (0.15)*	−0.06 (0.04)	−1.07 (0.2)*	−0.73 (0.16)*	−0.66 (0.14)*
Panic Disorder	−6.3 (0.86)*	−2.96 (0.73)*	−3.34 (0.45)*	53.06 (7.06)*	−0.33 (0.09)*	−0.39 (0.11)*	−0.05 (0.03)	−1.1 (0.17)*	−0.61 (0.13)*	−0.8 (0.14)*
Posttraumatic Stress	−5.16 (0.91)*	−0.83 (0.8)	−4.33 (0.59)*	84 (13.49)*	−0.43 (0.11)*	−0.79 (0.16)*	−0.1 (0.05)	−1.49 (0.22)*	−0.78 (0.16)*	−0.69 (0.15)*
Social Phobia	−3.07 (0.68)*	−2.01 (0.62)*	−1.06 (0.3)*	34.61 (9.81)*	−0.18 (0.06)*	−0.01 (0.07)	0.03 (0.02)	−0.32 (0.1)*	−0.27 (0.09)*	−0.26 (0.09)*
Specific Phobia	−2.26 (0.48)*	−1.67 (0.43)*	−0.59 (0.23)*	26.03 (8.92)*	−0.03 (0.02)	0 (0.06)	0.01 (0.01)	−0.22 (0.09)*	−0.19 (0.06)*	−0.15 (0.06)*
Headache/Migrane	−4.83 (0.34)*	−3.31 (0.31)*	−1.52 (0.14)*	31.43 (2.91)*	−0.12 (0.03)*	−0.08 (0.04)	−0.03 (0.01)*	−0.54 (0.06)*	−0.33 (0.05)*	−0.39 (0.05)*
Insomnia	−6.18 (0.52)*	−3.09 (0.47)*	−3.09 (0.25)*	49.99 (4.3)*	−0.21 (0.06)*	−0.64 (0.08)*	−0.07 (0.03)*	−1.03 (0.11)*	−0.56 (0.08)*	−0.52 (0.08)*
Neurological	−9.82 (1.12)*	−4.17 (0.93)*	−5.65 (0.78)*	57.55 (6.82)*	−0.37 (0.1)*	−1.45 (0.29)*	−0.24 (0.11)*	−1.58 (0.26)*	−0.84 (0.18)*	−1.06 (0.21)*
Arthritis	−5.47 (0.38)*	−3.46 (0.34)*	−2.01 (0.15)*	36.76 (2.77)*	−0.04 (0.01)*	−0.65 (0.07)*	−0.05 (0.02)*	−0.64 (0.07)*	−0.24 (0.04)*	−0.36 (0.05)*
Back/Neck Pain	−6.56 (0.31)*	−4.15 (0.28)*	−2.41 (0.13)*	36.77 (1.93)*	−0.07 (0.02)*	−0.62 (0.06)*	−0.04 (0.02)*	−0.95 (0.07)*	−0.32 (0.04)*	−0.4 (0.05)*
Cancer	−2.67 (0.86)*	−0.56 (0.76)	−2.11 (0.37)*	78.9 (22.6)*	−0.08 (0.05)	−0.64 (0.14)*	−0.05 (0.03)	−0.86 (0.15)*	−0.34 (0.09)*	−0.12 (0.08)
Cardiovascular	−5.8 (0.35)*	−3.59 (0.33)*	−2.22 (0.15)*	38.17 (2.75)*	−0.06 (0.02)*	−0.65 (0.07)*	−0.07 (0.03)*	−0.69 (0.07)*	−0.33 (0.05)*	−0.39 (0.05)*
Diabetes	−5.86 (0.65)*	−3.81 (0.57)*	−2.05 (0.27)*	35 (4.45)*	−0.11 (0.03)*	−0.59 (0.1)*	−0.07 (0.03)*	−0.81 (0.12)*	−0.25 (0.06)*	−0.21 (0.06)*
Digestive	−4.06 (0.59)*	−2.8 (0.52)*	−1.26 (0.26)*	31.01 (5.79)*	−0.01 (0.02)	−0.22 (0.08)*	−0.02 (0.01)	−0.47 (0.11)*	−0.24 (0.07)*	−0.29 (0.07)*
Respiratory	−1.49 (0.31)*	−1.2 (0.27)*	−0.29 (0.11)*	19.4 (6.72)*	−0.02 (0.01)	−0.1 (0.04)*	0 (0.01)	−0.17 (0.05)*	−0.02 (0.03)	0.01 (0.03)
**Direct effects of scales**	Cognition: −0.12 (0.03)* Mobility: −0.16 (0.01)* Self-care: −0.05 (0.02)* Getting along: −0.01 (0.03)Role functioning: −0.12 (0.01)* Family burden: −0.11 (0.01)* Stigma: −0.11 (0.01)* Discrimination: −0.02 (0.02)									

WMH surveys.

p-value<0.05.

1 Only dimensions with statistically significant effect are included. Getting along and Discrimination not statistically significant.

**Figure 3:**
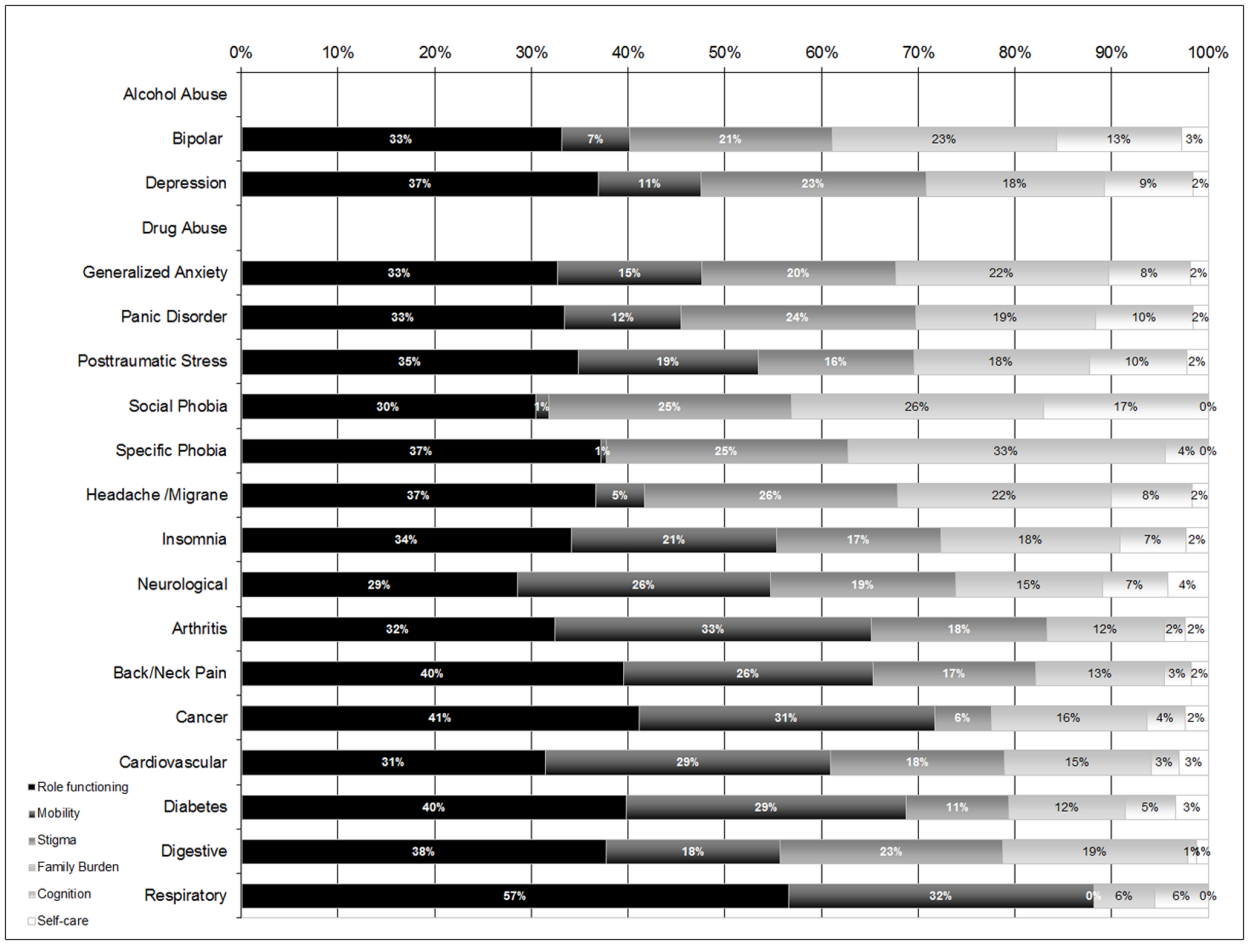
Relative WHODAS dimension contributions to the indirect effect of disability on perceived health VAS for each condition, overall sample. WMH Surveys (Alcohol Abuse and Drug Abuse are not represented because their respective overall indirect effect is not significant).

Figure 3 shows the relative importance of each disability dimension (adjusted by the rest of dimensions and comorbidity) in the disorder indirect effects on VAS scores. Thus, the 100% is the overall indirect effect of each condition, and the sections correspond to the different disability dimensions. Only the conditions with significant overall indirect effects are considered. It can be observed that role functioning is the most important mediator for all of the conditions with the exception of arthritis. The contribution of role functioning to the overall indirect effects ranges from 29% to 57%, and tends to be a bit more important among physical (median 36.5%, IQR = 32.4 to 39.3) than among mental conditions (median 32.9%, IQR = 32.0 to 35.2).

The differential disability mediation pattern for physical and mental conditions is evident for mobility, with a median of 27.2% of indirect effects for physical conditions versus 10.3% for mental. Conversely, stigma and family burden tend to be more important mediators of perceived health for mental conditions (medians for stigma are 22.7% for mental and 17.2% for physical, and for family burden, 21.7% and 15.0%, respectively). Cognition and self care have a low contribution to the indirect effects (median across all conditions 6.6% and 1.85%, respectively).

Tables S1, S2, and S3 are equivalent to Table 4 but restricting the sample to each of the three income country level groups. While all the total effects are statistically significant in the whole sample, not all of them are significant at the income country level. Posttraumatic stress has the highest proportion of indirect over total effects across mental conditions in the three groups. Neurological has the highest proportion for low/lower-middle and upper-middle income countries across physical conditions, while the corresponding one for high income countries is cancer.

The contributions of overall indirect to total effects among the nineteen conditions are shown in column 4 of each table of supporting information. These contributions range (among significant proportions): for high income countries, from 33.5% to 90.6%, with a median of 42.6% (IQR = 35.2–54.5); for upper-middle income countries, from 16.7% to 61.1%, median 33.2% (IQR = 26.5–48.6), and for low/lower-middle income countries, from 20.5% to 122.6%, median 38.7%, (IQR = 25.8–45.9). Hence, high income countries show the highest indirect, disability meditated, contribution.

Income country level information corresponding to Figure 2 (the proportion of indirect specific over the overall indirect effect) indicate that in all income groups, mobility has a higher contribution in physical conditions, and the indirect effects of family burden and stigma are higher among mental conditions. While role functioning is also the most important dimension for high and low/lower-middle income countries (median percentages and IQRs are 36.2 (32.9–40.8) and 35.9 (30.0–39.0), respectively), for middle-income countries it is stigma: median percentage 38.8, IQR = (31.4–45.1). (Data not shown but available on request.)

## Discussion

In this international study, we found that over a third (median of 36.8% with an IQR of 31.2% to 51.5%) of the total decrement in perceived health associated with common conditions is mediated by the disabilities assessed in the WHODAS. The magnitude of this mediated effect is exactly the same for mental disorders and for physical conditions. We also found that role functioning is the predominant dimension which indirectly accounts for the association of all the conditions with perceived health. While mobility is the second most important mediator in the case of chronic physical conditions, for mental conditions stigma and family burden are more important mediators. These results are not only statistically significant, but substantially relevant given the size and the consistency of the associations found. Taken together, these results confirm our a priori hypotheses suggesting that disability dimensions mediate the decrease of perceived health associated to chronic conditions and that the mediating dimensions are different for mental and physical conditions. Of notice these results are very similar across the three levels of country income. The fact that more than one third of perceived health decrements associated with common conditions is attributable to disability dimensions should call attention of the potential interest of assessing disability as well as to try to improve it, to ameliorate the health status of individuals with chronic conditions. That would require a systematic evaluation of disability dimensions in order to identify potential beneficial interventions.

To our knowledge, the mediating role of disability on perceived health has never been reported for samples of the general population representing so many countries worldwide for the large range of conditions and disability dimensions assessed here. Nevertheless, many studies have previously shown an association between particular dimensions of disability and perceived health, in samples of patients with particular diseases. For instance, social functioning is an important determinant of perceived health among heart failure patients [40] while physical ability has an important role on perceived health among spinal cord injury patients [41]. And for individuals with major depressive episode, cognition and embarrassment seem to be more relevant disability dimensions [30]. Our results represent a first systematic attempt to disentangle the association between a range of chronic conditions and perceived health considering a comprehensive range of disability indicators. And they indicate that, on average, the disability mediated effect on perceived health is substantial and similar for the 9 mental conditions and the 10 physical conditions analyzed. Nevertheless, the type of disability dimension which mediates such effect tends to be different for physical and for mental conditions. Moreover, there is variation across individual disorders in the extent to which their impact on perceived health is mediated by disability dimensions. More research is needed for further understanding the underlying process of perceived health and disability evaluations and how they may differ by different levels of health.

Mobility disability is a frequent mediator of the effect of chronic physical conditions on perceived health (median value of 10.2% of the total effect), while this dimension is much less important for mental conditions (3.2%). Many of the physical conditions considered in our study imply either pain (arthritis, back-neck pain) or impairment on the extremities and their functional performance (neurological conditions, cardiovascular, respiratory), or general weakness (cancer and others). All of which have an impact on the mobility function and modify the perception of health of the individual [13], [17] [42]. On the other hand, this disability dimension is not a very relevant mediator of the impact of mental conditions on perceived health, while family burden and stigma are. The empirical direct and indirect associations described here provide a textured picture of the ways health conditions impact on health perceptions and the role of functioning and disability. This might be important beyond description and might help guiding therapeutic efforts towards particular disabilities. For instance, in a descriptive study of breast cancer survivors it was estimated that potential interventions including physical mobility could prevent decreases in self-rated health among breast cancer survivors [43]. Also, the use of specific clinical problem-solving tools for physical and rehabilitation medicine could be liaised with assessments of perceived health [44]. Consistent with previous work [30], our data suggest that assessing stigma and family burden and trying to combat them can limit the decrements in perceived health of individuals with mental conditions. The type of relationships described here for the general adult population suggests that a systematic assessment of disability might help identifying areas of needed improvement for individuals with chronic conditions. Our results also suggest that effectively addressing disability should have a noticeable positive impact on the overall perception of health of the general population.

One remarkable finding of this study is the consistency of results across income country levels. We did find differences in the prevalence of disability: individuals in high income countries were twice as much likely to endorse any WHODAS disability than those in upper-middle or low and lower-middle income countries. These differences are consistent with previous reports in the literature indicating that cultural and work-related issues as well as differential access to health and social services could cause higher rates of disability in developed countries [45], [46]. In contrast, perceived health levels were very similar across countries (see Table 3), both in the general population and among individuals with chronic conditions. The proportion of perceived health accounted by disability is very consistent across income country levels, both for the overall WHODAS and for that of the specific dimensions. Only marginal departures were found in low and lower-middle income countries, with a higher frequency of non statistical significant associations, due in part to a smaller sample size. This substantial homogeneity across countries does not mean, on the other hand, that local culture can be ignored. The need to take into account ethnic, cultural, and social dimensions in combating disability [47]–[49] is well-established.

Our results must be interpreted taking into account the following limitations. First, chronic physical conditions and mental conditions were differently assessed. The latter were measured with a standard diagnostic instrument, the CIDI, with high levels of reliability and acceptable validity for research purposes. Conversely, chronic physical conditions were self-reported by respondents. Although we used standardized questions which have shown acceptable validity levels [32], [33], misclassification cannot be ruled out, in particular, underreporting of physical health conditions in countries with lower access to health care. Second, we did not assess some particularly disabling brain conditions such as non-affective psychotic conditions and dementia [50]. Our study, therefore, likely underestimates disability caused by mental conditions. Third, only 12-month physical and mental conditions were considered in this study, to increase the accuracy of recalls. Nevertheless, while physical conditions and mental conditions were assessed in the 12 months previous to the interview, both overall perceived health (VAS) and the WHODAS questions referred to the 30 days preceding the interview. Due to different time frames it is not possible to definitively relate either the health status nor the disability reported by the respondents to their underlying mental of physical health condition for the preceding 12 months. Nevertheless, because both, the VAS and the WHODAS use the same recall period, any such bias should not influence our analyses of the intermediating role of disability in the impact of conditions on perceived health. Similarly, we were not able to assess the duration of the disability. It has been suggested that age at disability onset may impact self-reported general health and should be considered when analyzing HRQOL differences within people with disabilities [51]. Finally, an important consideration is the difficulty to differentiate the nature of conditions, symptoms, function and perceptions, as well as the need to refine the mediating and/or moderating nature of the described associations [52].

### Implications

Our results, which are basically descriptive, call attention on the need to assess and consider disability to better understand how perceived health is influenced by common mental and physical conditions. More than a third of the decrements in perceived health are mediated by disability dimensions and would not be a direct effect of these conditions. This should call attention to the importance of addressing disability to increase health status among individual with common conditions. While disability can be more or less obviously related with the index condition, a systematic evaluation of disability could be beneficial. While role limitation and mobility are the disability most frequently mediating the effect of chronic physical conditions, stigma is an important mediator dimension for mental disorders. Measuring stigma among individuals with mental disorders should improve understanding of their perceived health reports. If the association of mental disorders and stigma is causal, combating stigma effectively could translate in gains in perceived health of individuals with mental disorders. Taken together, the findings described here suggest that there is need to learn more about the strength and ways of indirect association between chronic conditions and perceived health. In particular, evaluating whether interventions addressed to improve specific disabilities may improve perceived health of individuals with common chronic conditions beyond benefits that would be obtained with the usual treatment for these conditions.

## Supporting Information

Table S1 **Effects (direct and indirect via WHODAS) of conditions on perceived health VAS. WMH high income countries.** * p-value<0.05. ^1^ Only dimensions with statistically significant effect are included. Getting along and Discrimination not statistically significant.
(DOC)

Table S2 **Effects (direct and indirect via WHODAS) of conditions on perceived health VAS. WMH surveys middle income countries.** * p-value<0.05, ^1^ Only dimensions with statistically significant effect are included. Cognition, Self-care, Getting along and Discrimination not statistically significant.
(DOC)

Table S3 **Effects (direct and indirect via WHODAS) of conditions on perceived health VAS. WMH surveys low income countries.** * p-value<0.05, ^1^ Only dimensions with statistically significant effect are included. Self-care, Getting along and Discrimination not statistically significant.
(DOC)
