# Correction: Disability Mediates the Impact of Common Conditions on Perceived Health

**DOI:** 10.1371/annotation/1b1c6fa4-a665-4241-8cdc-fad6eb6709fc

**Published:** 2013-09-19

**Authors:** Jordi Alonso, Gemma Vilagut, Núria D. Adroher, Somnath Chatterji, Yanling He, Laura Helena Andrade, Evelyn Bromet, Ronny Bruffaerts, John Fayyad, Silvia Florescu, Giovanni de Girolamo, Oye Gureje, Josep Maria Haro, Hristo Hinkov, Chiyi Hu, Noboru Iwata, Sing Lee, Daphna Levinson, Jean Pierre Lépine, Herbert Matschinger, Maria Elena Medina-Mora, Siobhan O'Neill, J. Hormel, Jose A. Posada-Villa, Nezar Ismet Taib, Miguel Xavier, Ronald C. Kessler

An affiliation of the second author is missing. Gemma Vilagut is not only affiliated with institution number 1, but also institution number 3, CIBER en Epidemiología y Salud Pública, Barcelona, Spain.

In addition, the name of the 23rd author was given incorrectly. The correct name is: J. Hans Ormel. 

