# Supplementary material for: Disability Mediates the Impact of Common Conditions on Perceived Health
Source: PLoS One. 2013 Jun 6;8(6):e65858. doi: 10.1371/journal.pone.0065858 (PMC3675077; doi:10.1371/journal.pone.0065858)
Supplement: Table S1 — Effects (direct and indirect via WHODAS) of conditions on perceived health VAS. WMH high income countries. * p-value<0.05. 1 Only dimensions with statistically significant effect are included. Getting along and Discrimination not statistically significant. (DOC) [file pone.0065858.s001.doc]

Table S1. Effects (direct and indirect via WHODAS) of conditions on perceived health VAS. WMH high income countries.

|  |  |  |  |  | **Indirect effects via each WHODAS dimension^1^** | | | | | |
| --- | --- | --- | --- | --- | --- | --- | --- | --- | --- | --- |
|  | **Total effects of conditions on VAS** | **Direct effects of conditions** | **Indirect effects via WHODAS Scales** | **Proportion of indirect effects over total effects** | **Cognition** | **Mobility** | **Self-care** | **Role functioning** | **Family burden** | **Stigma** |
|  | **Coeff (SE)** | **Coeff (SE)** | **Coeff (SE)** | **% (SE)** | **Coeff (SE)** | **Coeff (SE)** | **Coeff (SE)** | **Coeff (SE)** | **Coeff (SE)** | **Coeff (SE)** |
| Alcohol Abuse | -1.48 (0.76) | -1.81 (0.69)* | 0.34 (0.37) | -22.98 (31.92) | 0.01 (0.04) | 0.38 (0.09)* | 0.05 (0.03)* | -0.13 (0.17) | 0.04 (0.08) | -0.03 (0.09) |
| Bipolar | -7.62 (1.51)* | -3.63 (1.23)* | -3.99 (1.01)* | 52.36 (11.08)* | -0.64 (0.19)* | -0.4 (0.26) | -0.11 (0.08) | -1.3 (0.33)* | -0.64 (0.23)* | -0.61 (0.21)* |
| Depression | -8.31 (0.54)* | -5.48 (0.52)* | -2.82 (0.29)* | 34 (3.46)* | -0.38 (0.09)* | -0.19 (0.09)* | -0.02 (0.02) | -1.18 (0.13)* | -0.44 (0.09)* | -0.49 (0.1)* |
| Drug Abuse | -2.61 (1.54) | -2.84 (1.28)* | 0.23 (0.65) | -8.91 (28.27) | 0 (0.08) | 0.3 (0.11)* | 0.01 (0.02) | -0.11 (0.29) | -0.03 (0.15) | 0.13 (0.14) |
| Generalized Anxiety | -5.19 (1.1)* | -2.38 (0.87)* | -2.81 (0.7)* | 54.12 (11.13)* | -0.35 (0.11)* | -0.37 (0.2) | -0.02 (0.03) | -0.93 (0.27)* | -0.56 (0.16)* | -0.46 (0.15)* |
| Panic Disorder | -6.09 (0.98)* | -2.92 (0.88)* | -3.17 (0.59)* | 51.99 (9.41)* | -0.46 (0.12)* | -0.47 (0.16)* | -0.09 (0.06) | -1.11 (0.22)* | -0.41 (0.13)* | -0.53 (0.15)* |
| Posttraumatic Stress | -4.87 (1)* | -0.95 (0.82) | -3.92 (0.79)* | 80.44 (14.68)* | -0.59 (0.17)* | -0.6 (0.21)* | -0.09 (0.07) | -1.46 (0.28)* | -0.58 (0.18)* | -0.47 (0.16)* |
| Social Phobia | -2.44 (0.81)* | -1.48 (0.78) | -0.96 (0.37)* | 39.25 (16.6)* | -0.25 (0.08)* | 0.06 (0.09) | 0.05 (0.03) | -0.28 (0.12)* | -0.26 (0.1)* | -0.22 (0.09)* |
| Specific Phobia | -2.75 (0.63)* | -1.83 (0.59)* | -0.92 (0.36)* | 33.48 (12.29)* | 0 (0.03) | -0.09 (0.11) | 0 (0.02) | -0.46 (0.16)* | -0.2 (0.08)* | -0.15 (0.07)* |
| Headache /Migraine | -4.12 (0.45)* | -2.62 (0.41)* | -1.5 (0.22)* | 36.4 (4.99)* | -0.16 (0.04)* | -0.14 (0.07) | -0.03 (0.02) | -0.61 (0.1)* | -0.3 (0.06)* | -0.2 (0.05)* |
| Insomnia | -6.16 (0.64)* | -2.78 (0.55)* | -3.38 (0.34)* | 54.88 (5.51)* | -0.25 (0.06)* | -0.85 (0.13)* | -0.11 (0.05)* | -1.13 (0.15)* | -0.52 (0.11)* | -0.4 (0.1)* |
| Neurological | -11.26 (1.41)* | -4.71 (1.22)* | -6.54 (1.26)* | 58.14 (9.06)* | -0.59 (0.17)* | -2.02 (0.48)* | -0.39 (0.21) | -1.75 (0.39)* | -0.65 (0.24)* | -0.84 (0.26)* |
| Arthritis | -4.89 (0.48)* | -2.81 (0.41)* | -2.08 (0.21)* | 42.58 (4.17)* | -0.05 (0.02)* | -0.87 (0.1)* | -0.08 (0.04)* | -0.7 (0.11)* | -0.14 (0.04)* | -0.23 (0.05)* |
| Back/Neck Pain | -6.69 (0.38)* | -3.31 (0.33)* | -3.38 (0.2)* | 50.51 (2.95)* | -0.12 (0.03)* | -0.97 (0.11)* | -0.08 (0.03)* | -1.41 (0.12)* | -0.38 (0.07)* | -0.36 (0.07)* |
| Cancer | -1.9 (0.91)* | -0.18 (0.81) | -1.72 (0.42)* | 90.62 (38.85)* | -0.11 (0.06) | -0.4 (0.16)* | -0.03 (0.03) | -0.73 (0.17)* | -0.29 (0.09)* | -0.13 (0.07) |
| Cardiovascular | -5.5 (0.45)* | -3.16 (0.4)* | -2.34 (0.24)* | 42.57 (4.13)* | -0.1 (0.03)* | -0.86 (0.11)* | -0.1 (0.06) | -0.7 (0.1)* | -0.28 (0.07)* | -0.25 (0.05)* |
| Diabetes | -5.72 (0.81)* | -3.44 (0.71)* | -2.28 (0.39)* | 39.85 (6.42)* | -0.11 (0.04)* | -0.72 (0.16)* | -0.1 (0.04)* | -0.92 (0.18)* | -0.18 (0.07)* | -0.19 (0.07)* |
| Digestive | -6.95 (1.1)* | -3.03 (0.97)* | -3.92 (0.5)* | 56.38 (8.34)* | -0.12 (0.06)* | -1.05 (0.2)* | -0.09 (0.05) | -1.47 (0.23)* | -0.52 (0.14)* | -0.57 (0.14)* |
| Respiratory | -0.55 (0.35) | -0.48 (0.31) | -0.07 (0.13) | 12.53 (21.17) | -0.03 (0.02) | -0.04 (0.05) | 0.01 (0.01) | -0.08 (0.06) | 0.03 (0.03) | 0.02 (0.02) |
| **Direct effects of scales** | Cognition: -0.15 (0.03)* Mobility: -0.17 (0.02)* Self-care: -0.06 (0.03)* Getting along: -0.02 (0.03)  Role functioning: -0.13 (0.01)* Family burden: -0.1 (0.02)* Stigma: -0.1 (0.02)* Discrimination: -0.04 (0.02) | | | | | | | | | |

* p-value < 0.05

^1^ Only dimensions with statistically significant effect are included. Getting along and Discrimination not statistically significant
