# Supplementary material for: Disability Mediates the Impact of Common Conditions on Perceived Health
Source: PLoS One. 2013 Jun 6;8(6):e65858. doi: 10.1371/journal.pone.0065858 (PMC3675077; doi:10.1371/journal.pone.0065858)
Supplement: Table S2 — Effects (direct and indirect via WHODAS) of conditions on perceived health VAS. WMH surveys middle income countries. * p-value<0.05, 1 Only dimensions with statistically significant effect are included. Cognition, Self-care, Getting along and Discrimination not statistically significant. (DOC) [file pone.0065858.s002.doc]

**Table S2. Effects (direct and indirect via WHODAS) of conditions on perceived health VAS. WMH surveys middle income countries**

|  |  |  |  |  | **Indirect effects via each WHODAS dimension^1^** | | | |
| --- | --- | --- | --- | --- | --- | --- | --- | --- |
|  | **Total effects of conditions on VAS** | **Direct effects of conditions** | **Indirect effects via WHODAS Scales** | **Proportion of indirect effects over total effects** | **Mobility** | **Role functioning** | **Family burden** | **Stigma** |
|  | **Coeff (SE)** | **Coeff (SE)** | **Coeff (SE)** | **% (SE)** | **Coeff (SE)** | **Coeff (SE)** | **Coeff (SE)** | **Coeff (SE)** |
| Alcohol Abuse | -5.89 (2.47)* | -5.07 (2.39)* | -0.82 (0.54) | 13.94 (9.56) | 0.15 (0.11) | -0.26 (0.15) | -0.2 (0.14) | -0.45 (0.26) |
| Bipolar | -6.45 (2.17)* | -2.56 (2.08) | -3.89 (0.97)* | 60.26 (21.07)* | 0.02 (0.11) | -0.63 (0.24)* | -1.32 (0.4)* | -1.83 (0.57)* |
| Depression | -8.29 (1.03)* | -4.81 (1.01)* | -3.48 (0.44)* | 41.95 (6.41)* | -0.48 (0.13)* | -0.92 (0.22)* | -0.73 (0.21)* | -1.21 (0.27)* |
| Drug Abuse | -0.25 (3.75) | 0.16 (3.32) | -0.41 (0.93) | 163.16 (2253.91) | -0.03 (0.26) | -0.02 (0.25) | -0.12 (0.21) | -0.26 (0.43) |
| Generalized Anxiety | -5.63 (2.29)* | -3.76 (2.25) | -1.87 (0.85)* | 33.22 (17.81) | -0.38 (0.2) | -0.56 (0.26)* | -0.3 (0.2) | -0.59 (0.43) |
| Panic Disorder | -7.78 (1.83)* | -3.77 (1.85)* | -4.01 (0.85)* | 51.53 (14.47)* | -0.31 (0.2) | -0.74 (0.22)* | -1.05 (0.35)* | -1.81 (0.54)* |
| Posttraumatic Stress | -5.24 (2.41)* | -2.04 (2.11) | -3.21 (1.08)* | 61.12 (25.5)* | -0.55 (0.34) | -0.56 (0.3) | -0.59 (0.27)* | -1.42 (0.42)* |
| Social Phobia | -5.01 (1.68)* | -3.17 (1.59)* | -1.84 (0.71)* | 36.75 (15.39)* | -0.15 (0.18) | -0.46 (0.22)* | -0.38 (0.18)* | -0.83 (0.37)* |
| Specific Phobia | -2.74 (0.9)* | -1.93 (0.83)* | -0.81 (0.36)* | 29.7 (12.91)* | -0.09 (0.11) | -0.21 (0.1)* | -0.16 (0.11) | -0.32 (0.15)* |
| Headache /Migraine | -4.98 (0.74)* | -4.15 (0.68)* | -0.83 (0.26)* | 16.68 (4.88)* | -0.05 (0.07) | -0.18 (0.08)* | -0.14 (0.07)* | -0.43 (0.13)* |
| Insomnia | -4.37 (1.18)* | -3.24 (1.07)* | -1.13 (0.43)* | 25.9 (9.24)* | -0.02 (0.09) | -0.15 (0.09) | -0.36 (0.13)* | -0.49 (0.22)* |
| Neurological | -6.91 (2.58)* | -2.87 (2.38) | -4.04 (1.02)* | 58.47 (21.05)* | -1.24 (0.48)* | -1.1 (0.29)* | -0.78 (0.33)* | -0.89 (0.47) |
| Arthritis | -5.7 (0.85)* | -3.54 (0.82)* | -2.16 (0.26)* | 37.85 (6.22)* | -0.42 (0.1)* | -0.32 (0.08)* | -0.36 (0.1)* | -1.01 (0.18)* |
| Back/Neck Pain | -6.85 (0.79)* | -4.99 (0.7)* | -1.86 (0.27)* | 27.17 (3.66)* | -0.56 (0.13)* | -0.5 (0.11)* | -0.21 (0.08)* | -0.58 (0.15)* |
| Cancer | -4.93 (2.86) | -2.68 (2.15) | -2.25 (1.13)* | 45.69 (18.01)* | -0.73 (0.42) | -0.55 (0.36) | -0.44 (0.33) | -0.53 (0.47) |
| Cardiovascular | -7.28 (0.74)* | -5.24 (0.77)* | -2.04 (0.22)* | 27.99 (4.12)* | -0.39 (0.09)* | -0.51 (0.1)* | -0.37 (0.11)* | -0.73 (0.18)* |
| Diabetes | -5.54 (1.54)* | -4.51 (1.38)* | -1.04 (0.45)* | 18.71 (7.28)* | -0.38 (0.18)* | -0.38 (0.14)* | -0.07 (0.1) | -0.23 (0.2) |
| Digestive | -1.81 (1.23) | -1.22 (1.25) | -0.6 (0.5) | 32.92 (32.66) | 0.07 (0.13) | -0.12 (0.13) | -0.12 (0.14) | -0.41 (0.24) |
| Respiratory | -3.08 (0.98)* | -3.3 (0.92)* | 0.22 (0.24) | -7.2 (8.82) | 0.12 (0.08) | 0.1 (0.07) | 0.01 (0.05) | 0 (0.11) |
| **Direct effects of scales** | Cognition: -0.02 (0.07) Mobility: -0.16 (0.03)* Self-care: 0.01 (0.05) Getting along: 0.02 (0.07)  Role functioning: -0.08 (0.01)* Family burden: -0.1 (0.03)* Stigma: -0.16 (0.03)* Discrimination: -0.02 (0.03) | | | | | | | |

* p-value < 0.05

^1^ Only dimensions with statistically significant effect are included. Cognition, Self-care, Getting along and Discrimination not statistically significant
