# Supplementary material for: Disability Mediates the Impact of Common Conditions on Perceived Health
Source: PLoS One. 2013 Jun 6;8(6):e65858. doi: 10.1371/journal.pone.0065858 (PMC3675077; doi:10.1371/journal.pone.0065858)
Supplement: Table S3 — Effects (direct and indirect via WHODAS) of conditions on perceived health VAS. WMH surveys low income countries. * p-value<0.05, 1 Only dimensions with statistically significant effect are included. Self-care, Getting along and Discrimination not statistically significant. (DOC) [file pone.0065858.s003.doc]

**Table S3. Effects (direct and indirect via WHODAS) of conditions on perceived health VAS. WMH surveys low income countries**

|  |  |  |  |  | **Indirect effects via each WHODAS dimension^1^** | | | | |
| --- | --- | --- | --- | --- | --- | --- | --- | --- | --- |
|  | **Total effects of conditions on VAS** | **Direct effects of conditions** | **Indirect effects via WHODAS Scales** | **Proportion of indirect effects over total effects** | **Cognition** | **Mobility** | **Role functioning** | **Family burden** | **Stigma** |
|  | **Coeff (SE)** | **Coeff (SE)** | **Coeff (SE)** | **% (SE)** | **Coeff (SE)** | **Coeff (SE)** | **Coeff (SE)** | **Coeff (SE)** | **Coeff (SE)** |
| Alcohol Abuse | -3.2 (1.37)* | -3.1 (1.29)* | -0.1 (0.42) | 3.21 (12.59) | 0.02 (0.04) | 0.15 (0.08) | -0.14 (0.19) | -0.12 (0.15) | -0.06 (0.12) |
| Bipolar | -10.83 (3.98)* | -7.52 (2.64)* | -3.31 (2.07) | 30.58 (12.2)* | -0.43 (0.34) | -0.08 (0.26) | -1.66 (0.82)* | -0.52 (0.37) | -0.36 (0.51) |
| Depression | -7.76 (0.87)* | -4.82 (0.77)* | -2.94 (0.47)* | 37.88 (5.55)* | -0.16 (0.1) | -0.44 (0.18)* | -0.92 (0.18)* | -0.56 (0.15)* | -0.7 (0.18)* |
| Drug Abuse | -11.93 (4.63)* | -7.36 (5.12) | -4.57 (2.14)* | 38.32 (23.42) | -0.57 (0.39) | -0.02 (0.33) | -1.14 (0.63) | -1.94 (1.05) | -1.12 (0.64) |
| Generalized Anxiety | -4.94 (2.19)* | 0.39 (1.7) | -5.33 (1.34)* | 107.93 (37.32)* | -0.37 (0.26) | -0.61 (0.35) | -1.54 (0.43)* | -1.47 (0.54)* | -1.04 (0.33)* |
| Panic Disorder | -5.58 (2.31)* | -2.3 (1.75) | -3.28 (1.12)* | 58.81 (18.04)* | -0.28 (0.23) | -0.25 (0.18) | -1.22 (0.48)* | -0.68 (0.33)* | -0.83 (0.29)* |
| Posttraumatic Stress | -5.88 (2.71)* | 1.33 (2.72) | -7.21 (1.12)* | 122.58 (55.97)* | -0.58 (0.22)* | -1.29 (0.38)* | -2.25 (0.56)* | -1.77 (0.46)* | -1.08 (0.41)* |
| Social Phobia | -2.57 (1.68) | -2.01 (1.18) | -0.56 (0.87) | 21.61 (24.77) | -0.27 (0.22) | 0.07 (0.13) | 0 (0.29) | -0.21 (0.26) | -0.2 (0.17) |
| Specific Phobia | -0.95 (1.08) | -1.05 (0.94) | 0.1 (0.35) | -9.97 (44.33) | -0.07 (0.05) | 0.16 (0.07)* | 0.15 (0.12) | -0.19 (0.13) | -0.01 (0.1) |
| Headache /Migraine | -5.75 (0.68)* | -3.37 (0.63)* | -2.38 (0.26)* | 41.45 (5.32) | -0.19 (0.09)* | -0.22 (0.08)* | -0.91 (0.13)* | -0.54 (0.13)* | -0.46 (0.13)* |
| Insomnia | -7.82 (1.28)* | -3.88 (1.17)* | -3.94 (0.58)* | 50.39 (8.54)* | -0.16 (0.1) | -0.49 (0.17)* | -1.64 (0.29)* | -0.78 (0.23)* | -0.81 (0.2)* |
| Neurological | -8.92 (2.32)* | -3.97 (1.78)* | -4.96 (1.34)* | 55.55 (12.51)* | -0.21 (0.17) | -0.58 (0.34) | -1.43 (0.57)* | -1.26 (0.4)* | -1.38 (0.51)* |
| Arthritis | -6.2 (0.81)* | -4.34 (0.73)* | -1.86 (0.32)* | 30 (5)* | -0.1 (0.04)* | -0.42 (0.12)* | -0.71 (0.15)* | -0.35 (0.1)* | -0.26 (0.11)* |
| Back/Neck Pain | -5.92 (0.6)* | -4.71 (0.55)* | -1.21 (0.18)* | 20.46 (2.9)* | -0.03 (0.02) | -0.19 (0.06)* | -0.48 (0.09)* | -0.2 (0.06)* | -0.3 (0.08)* |
| Cancer | -8.77 (4.07)* | -5.32 (3.91) | -3.45 (1.61)* | 39.39 (21.73) | 0.04 (0.07) | -0.81 (0.51) | -1.42 (0.61)* | -0.45 (0.31) | -0.58 (0.4) |
| Cardiovascular | -4.93 (0.8)* | -2.96 (0.74)* | -1.97 (0.26)* | 39.94 (6.61)* | -0.05 (0.05) | -0.39 (0.11)* | -0.71 (0.12)* | -0.36 (0.1)* | -0.43 (0.12)* |
| Diabetes | -6.36 (1.42)* | -3.9 (1.31)* | -2.46 (0.56)* | 38.66 (9.67)* | -0.21 (0.11) | -0.26 (0.14) | -0.86 (0.24)* | -0.7 (0.25)* | -0.38 (0.15)* |
| Digestive | -2.04 (0.76)* | -2.87 (0.68)* | 0.83 (0.28)* | -40.77 (24.84) | 0.07 (0.05) | 0.23 (0.07)* | 0.24 (0.14) | 0.11 (0.11) | 0.14 (0.09) |
| Respiratory | -2.47 (0.64)* | -1.48 (0.56)* | -0.99 (0.29)* | 40.2 (11.37)* | -0.02 (0.03) | -0.16 (0.07)* | -0.47 (0.13)* | -0.21 (0.1)* | -0.12 (0.07) |
| **Direct effects of scales** | Cognition: -0.14 (0.06)* Mobility: -0.14 (0.03)* Self-care: -0.06 (0.04) Getting along: -0.03 (0.05)  Role functioning: -0.13 (0.01)* Family burden: -0.15 (0.03)* Stigma: -0.1 (0.02)* Discrimination: 0.02 (0.03) | | | | | | | | |

* p-value < 0.05
^1^ Only dimensions with statistically significant effect are included. Self-care, Getting along and Discrimination not statistically significant
